# Rotavirus-Induced Expansion of Antigen-Specific CD8 T Cells Does Not Require Signaling *via* TLR3, MyD88 or the Type I Interferon Receptor

**DOI:** 10.3389/fimmu.2022.814491

**Published:** 2022-04-07

**Authors:** Konjit Getachew Muleta, Isabel Ulmert, Kedir Hussen Hamza, Sharné van Dijl, Joy Nakawesi, Katharina Lahl

**Affiliations:** ^1^ Immunology Section, Lund University, Lund, Sweden; ^2^ Section for Experimental and Translational Immunology, Institute for Health Technology, Technical University of Denmark (DTU), Kongens Lyngby, Denmark

**Keywords:** rotavirus, CD8 T cells, dendritic cells, type I IFN, innate immunity, pattern (re)cognition

## Abstract

Rotavirus (RV) infection induces strong adaptive immunity. While protection from reinfection requires humoral immunity, initial clearance of infection depends on cytotoxic CD8 T cells. Type I classical dendritic cells (cDC1) excel at CD8 T cell induction through cross-presentation and are essential for optimal cytotoxicity towards RV. Upon sensing of infection-induced innate immune signals through pattern recognition receptors (PRRs), cumulating in autocrine type I interferon (IFN) signaling, cDC1 mature and migrate to the draining lymph nodes (LNs), where they prime adaptive immune cells. To analyze which PRR pathways lead to robust cytotoxicity in the context of RV infection, we measured RV-specific CD8 T cell priming in mice deficient for Toll-like receptor 3 (TLR3), recognizing double-stranded RNA, or for MyD88, the adapter for all other TLRs and IL-1 family cytokines. Individual TLR3- and MyD88-mediated signaling was not required for the priming of CD8 T cell responses to RV and neither deficiency impacted on RV clearance. Surprisingly, the accumulation of RV-specific CD8 T cells was also not altered in the absence of type I IFN signaling, while their ability to produce IFNγ and granzyme were blunted. Together, this suggests a substantial level of redundancy in the sensing of RV infection and the translation of signals into protective CD8 T cell immunity.

## Introduction

Rotavirus (RV) is a double-stranded (ds) enteric RNA virus with high epithelial cell tropism that causes severe dehydrating diarrhea in young children (1). Both natural infection and vaccination generally elicit strong adaptive immunity, which is essential for clearance of primary infection as well as protection from reinfection. While the innate immune signaling pathways leading to the control of the initial viral replication have been dissected in detail (2), the requirements for the induction of adaptive immunity to RV remain less well understood.

Dendritic cells (DCs) are essential for the induction of adaptive immunity. Classical DCs (cDCs) are broadly divided into two different subsets, cDC1 and cDC2. cDC1 excel at orchestrating immunity towards viral infection, intracellular pathogens, and tumors, which is assigned to their ability to cross-present antigen to CD8 T cells (3) and to their efficiency in inducing TH1 cells (4, 5). Accordingly, mice that lack cDC1 due to a deficiency in the transcription factor BATF3 show delayed clearance of RV, accompanied by significantly blunted RV-specific CD8 T cell responses (6, 7) and delayed induction of RV-specific IgA in the intestine-draining mesenteric lymph nodes (MLN) (7).

Viral recognition occurs through several pathways and pattern recognition receptors (PRRs) play important roles in the early innate response to viruses (8). The RIG-I/MDA5/MAVS, the TLR3/TRIF and the TLR7/MyD88 pathway were all implied in recognition of RV, cumulating in the induction of type I interferon (IFN). Type I IFN signaling through the type 1 IFN receptor (IFNAR) triggers the expression of an array of IFN-stimulated genes, which together coordinate the antiviral response, including both direct suppression of viral replication as well as modulation of the immune response (9–11). The high infectivity of homologous RV is assigned to its efficiency in suppressing signaling downstream of the type I IFN receptor in a host-specific manner, allowing the virus to replicate in the intestinal epithelium (12, 13) despite initial induction of type I IFNs (12). The role that type I IFN signaling plays in the induction of adaptive immunity is complex and includes direct effects on lymphocytes as well as indirect effects through DCs. Either a combination of type I IFN and IFNγ or IL-12 alone can act as a third signal in addition to TCR-engagement and co-stimulation to induce effector function and memory formation in CD8 T cells in several settings in a context dependent manner (14–17). The role of type I IFN in inducing adaptive cellular immunity towards RV has not been dissected in detail.

We here addressed the overall requirements for major innate sensing pathways previously implied in RV recognition by measuring RV-specific CD8 T cell induction in TLR3 and MyD88 deficient mice and found that they are not individually required for the induction of cellular adaptive immunity in adult mice. The lack of type I IFN sensing equally did not affect the quantity of RV-specific CD8 T cell induction despite the relative loss of RV-induced DC activation and migration to the draining LNs and a diminished bystander effector CD8 T cell response, along with a functional defect in the responding CD8 T cell compartment reflected in their lower production of IFNγ and granzyme A. Our data suggest a surprising level of redundancy across innate immune sensing pathways driving the initial proliferation of virus-specific cytotoxic CD8 T cells in the context of natural infection of adult mice with RV.

## Results

### TLR3 Is Not Required for the Generation of RV-Specific CD8 T Cell Responses in Adult Mice

Clearance of RV infection is mediated by cytotoxic CD8 T cells responses (18). DCs are required for the initiation of adaptive immune responses, where BATF3-dependent cDC1 play a crucial role in orchestrating cytotoxic immunity due to their unique ability to cross-present antigen to CD8 T cells. We and others have previously shown that the RV-specific CD8 T cell response is significantly blunted in the absence of cDC1 (6, 7). Rotavirus is a double-stranded (ds) RNA virus. Differences in the expression level of the dsRNA pattern recognition receptor (PRR) TLR3 between neonates and adults have previously been discussed to account for the pronounced susceptibility to RV infection by neonates (19). cDC1 uniquely express high levels of TLR3 (20), leading us to hypothesize that optimal CD8 T cell priming in response to RV infection may require dsRNA sensing through TLR3.

**Figure 1:**
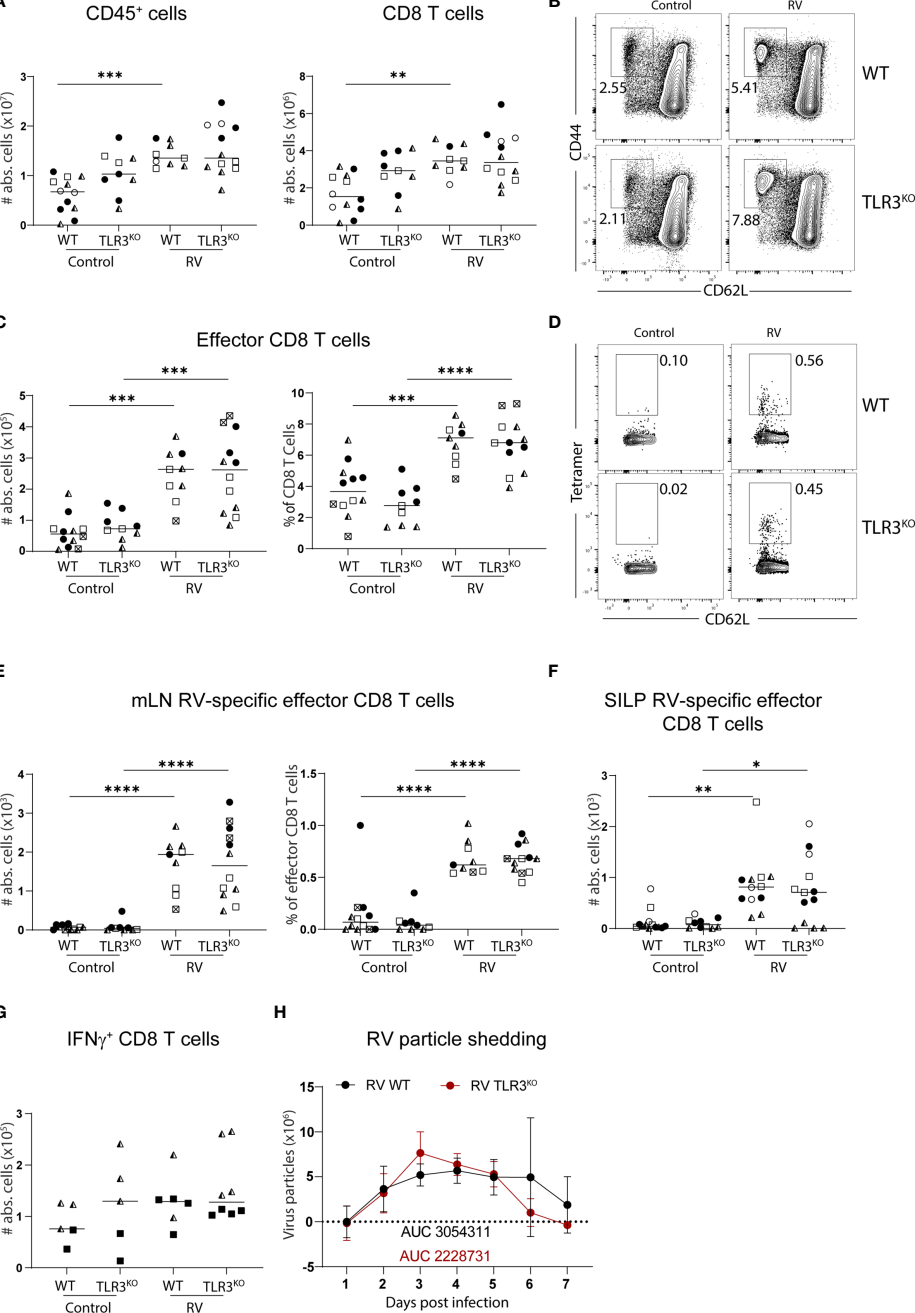
Lack of TLR3 does not affect the RV-specific CD8 T cell response. WT and TLR3^KO^ mice were infected with RV. **(A–E**, **G)** MLN cells and **(F)** SILP cells isolated from uninfected control and RV infected WT and TLR3^KO^ mice seven days post oral infection. **(A)** Total number of CD45^+^ cells and CD8 T cells (CD45^+^CD3^+^CD4^-^CD8α^+^). **(B)** Representative FACS plot of effector CD8 T cells (CD62L^-^CD44^+^) pre-gated on CD45^+^CD3^+^CD4^-^CD8α^+^ cells. **(C)** Absolute cell numbers and frequency of effector CD8 T cells (CD62L^-^CD44^+^). **(D)** Representative FACS plot of RV-specific effector CD8 T cells (CD44^+^CD62L^-^Tet^+^). **(E)** Absolute cell numbers and frequency of RV-specific effector CD8 T cells. **(F)** RV-specific CD8 T cells (CD45^+^CD3^+^CD4^-^CD8α^+^CD44^+^Tet^+^). **(G)** MLN cells isolated seven days post infection and restimulated with PMA/Ionomycin. Absolute cell number and frequency of IFNγ^+^ CD8 T cells (CD45^+^CD3^+^CD4^-^CD8α^+^CD44^+^IFNγ^+^) analysed by flow cytometry. **(H)** Level of RV shedding measured by ELISA on fecal samples collected at indicated time points (AUC, area under the curve). Each data point represents one mouse from a total of four different experiments (two for **(G)**, indicated by the different symbols. Ordinary one-way ANOVA with Tukey´s multiple comparison test was performed for statistical analysis. *p < 0.05, **p < 0.01, ***p < 0.001, ****p < 0.0001.

To assess the role of TLR3 in the induction of RV-specific CD8 T cell response in adult mice, we infected TLR3-deficient mice with the wildtype RV strain EC_W_ and analyzed the CD8 T cell compartment in the MLN seven days later by flow cytometry. In line with general MLN hypertrophy in response to RV infection (21), overall cellularity and CD8 T cell numbers were increased upon infection, and this was not affected by the absence of TLR3-signaling (**Figure 1A**). As expected, RV infection resulted in the accumulation of CD62L^-^CD44^+^ effector CD8 T cells in the RV-infected mice, which again did not depend on TLR3 (**Figures 1B, C**). Finally, we measured the RV-specific CD8 T cell response by using tetramers containing the VP6_VGPVFPPGM_ immunodominant peptide and found that priming of RV-specific CD8 T cells occurred in both wildtype (WT) and TLR3-deficient mice to a similar degree (**Figures 1D, E**). RV-specific effector CD8 T cell numbers were also not affected in the small intestinal lamina propria (SILP) (**Figure 1F**) and the slightly increased fraction of MLN CD8 T cells able to produce IFNγ in the context of RV infection was comparable between TLR3-deficient mice and WT controls (**Figure 1G**). Taken together, the absence of TLR3-signaling does not affect the priming of RV-specific CD8 T cells. Accordingly, fecal shedding of RV as a measure of infectious load was similar between WT and TLR3-deficient mice (**Figure 1H**), suggesting that the primed CD8 T cells in the absence of TLR3 were functional and that TLR3 is dispensable for early initiation events of cytotoxic immunity to RV.

### RV-Specific CD8 T Cell Accumulation Is Unaltered in MyD88-Deficient Adult Mice

**Figure 2:**
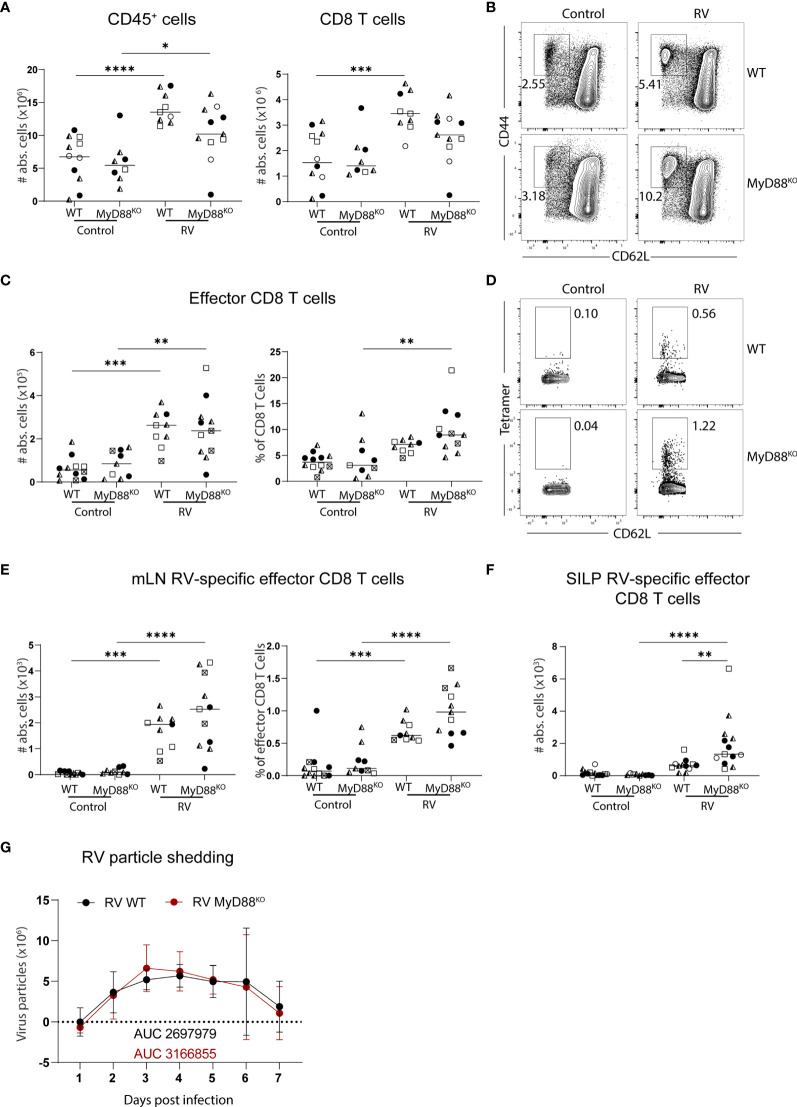
Lack of MyD88 signalling does not impact on the generation of the RV-specific CD8 T cell response. WT and MyD88^KO^ mice were infected with RV. **(A-E, G)** MLN cells and **(F)** SILP cells isolated from uninfected control and RV infected WT and MyD88^KO^ mice seven days post oral infection. **(A)** Total number of CD45^+^ cells and CD8 T cells (CD45^+^CD3^+^CD4^-^CD8α^+^). **(B)** Representative FACS plot of effector CD8 T cells (CD62L^-^CD44^+^) pre-gated on CD45^+^CD3^+^CD4^-^CD8α^+^ cells. **(C)** Absolute cell numbers and frequency of effector CD8 T cells (CD62L^-^CD44^+^). **(D)** Representative FACS plot of RV-specific effector CD8 T cells (CD44^+^CD62L^-^Tet^+^). **(E)** Absolute cell numbers and frequency of RV-specific effector CD8 T cells. **(F)** RV-specific CD8 T cells (CD45^+^CD3^+^CD4^-^CD8α^+^CD44^+^Tet^+^). **(G)** Level of RV shedding measured by ELISA on fecal samples collected at indicated time points. Each data point represents one mouse from a total of four different experiments, indicated by the different symbols. Ordinary one-way ANOVA with Tukey´s multiple comparison test was performed for statistical analysis. *p < 0.05, **p < 0.01, ***p < 0.001, ****p < 0.0001.

The adapter molecule myeloid differentiation primary response 88 (MyD88) orchestrates key pathways of innate immunity by mediating signaling downstream of the IL-1 receptor and all TLRs except for TLR3. MyD88-deficiency leads to susceptibility to a plethora of pathogens spanning bacteria, viruses, parasites and fungi (22). In the context of RV infection, MyD88-signaling is required for optimal humoral immunity, supporting both RV-specific IgA induction and an adequate IgG2c over IgG1 ratio, suggesting that MyD88-signaling supports TH1 responses (23). To determine whether MyD88 was also required for RV-specific CD8 T cell responses, we analyzed total and RV-specific effector CD8 T cells in the MLN of adult mice seven days post-infection. MLN hypertrophy and accordingly accumulation of total CD8 T cell numbers were comparable between MyD88-deficient and control mice (**Figure 2A**). Further, the generation of effector CD8 T cells after RV infection was not significantly altered (**Figures 2B, C**). Lastly, the priming of RV-specific CD8 T cells was not impaired by the absence of MyD88 signaling, and we instead detected a non-significant increase of CD8 T cells binding the RV tetramer (**Figures 2D, E**). This effect was more pronounced at the effector site, as MyD88-deficient mice harbored significantly increased numbers of RV-specific CD8 T cells in the SILP than WT controls (**Figure 2F**). We reasoned that elevated priming of RV-specific CD8 T cells may be driven by higher viral loads in the absence of MyD88 signaling (23), but viral shedding in our cohort was comparable between MyD88-deficient and control animals (**Figure 2G**). Taken together, MyD88 is dispensable for the induction of CD8 T cell priming in response to RV infection.

### cDC1 Can Prime RV-Specific CD8 T Cell Responses in the Absence of Type I IFN Sensing

**Figure 3:**
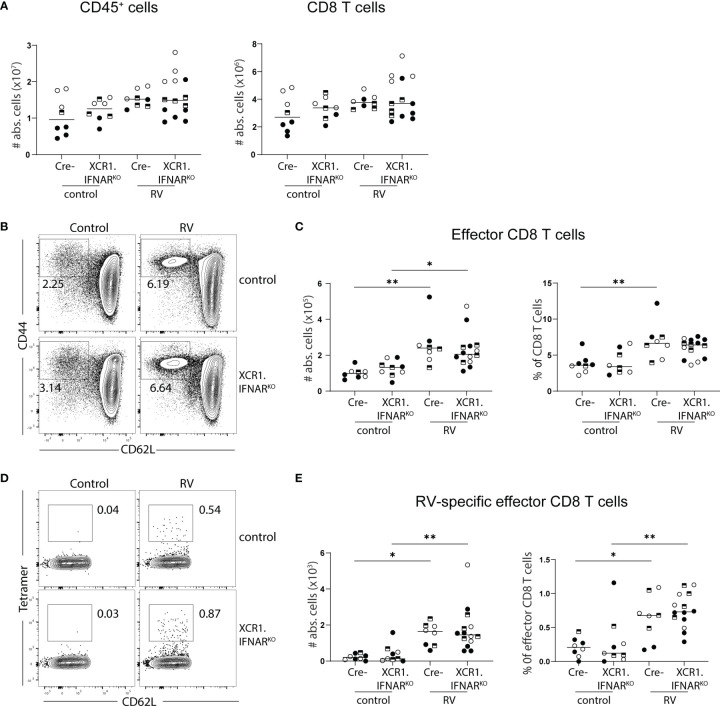
Interferon signalling *via* cDC1 is dispensable for the generation of the RV-specific CD8 T cell response. XCR1.IFNAR^KO^ and control mice were infected with RV. **(A-E)** MLN cells isolated from uninfected control and RV infected WT and XCR1.IFNAR ^KO^ mice seven days post oral infection. **(A)** Total number of CD45^+^ cells and CD8 T cells (CD45^+^CD3^+^CD4^-^CD8α^+^). **(B)** Representative FACS plot of effector CD8 T cells (CD62L^-^CD44^+^) pre-gated on CD45^+^CD3^+^CD4^-^CD8α^+^ cells. **(C)** Absolute cell numbers and frequency of effector CD8 T cells (CD62L^-^CD44^+^). **(D)** Representative FACS plot of RV-specific effector CD8 T cells (CD44^+^CD62L^-^Tet^+^). **(E)** Absolute cell numbers and frequency of RV-specific effector CD8 T cells. Each data point represents one mouse from a total of three different experiments, indicated by the different symbols. Ordinary one-way ANOVA with Tukey´s multiple comparison test was performed for statistical analysis. *p < 0.05, **p < 0.01.

Several PRRs can sense viral infections, explaining the redundancy of individual sensing pathways. Downstream signaling cumulates in the induction of type I IFNs, which were shown to orchestrate gene expression profile changes within DCs upon PRR sensing in an autocrine manner (24). We have previously shown that cDC1 depend on the ability to sense type I IFN for migration and activation in response to poly(I:C) (20). Furthermore, microbiota-induced steady-state type I IFN signaling in the intestines poises DCs into an immune inductive state, while the absence of type I IFN sensing renders intestinal DCs unable to prime effector T cells (25). Since cDC1 drive CD8 T cell priming in the context of RV infection (6, 7), we hypothesized that RV-specific CD8 T cell accumulation in the MLN would be diminished if cDC1 were unable to sense type I IFN. To test this, we generated mice lacking the type I IFN receptor specifically on cDC1 by crossing mice expressing cre recombinase under the control of the XCR1 promoter to mice carrying floxed alleles of IFNAR, hereafter called XCR1.IFNAR^KO^. Seven days after oral infection with RV, XCR1.IFNAR1^KO^ mice showed comparable increases of total CD45^+^ and CD8 T cell numbers to cre negative littermate controls (**Figure 3A**). Likewise, effector cell differentiation (**Figures 3B, C**) and priming of RV-specific CD8 T cells (**Figures 3D, E**) occurred to similar levels in XCR1.IFNAR1^KO^ and control mice. Thus, cDC1 can prime CD8 T cell responses to RV in the absence of cDC1-intrinsic type I IFN signaling.

### Global Type I IFNAR Deficiency Does Not Affect RV-Specific CD8 T Cell Abundancy in Adult Mice

**Figure 4:**
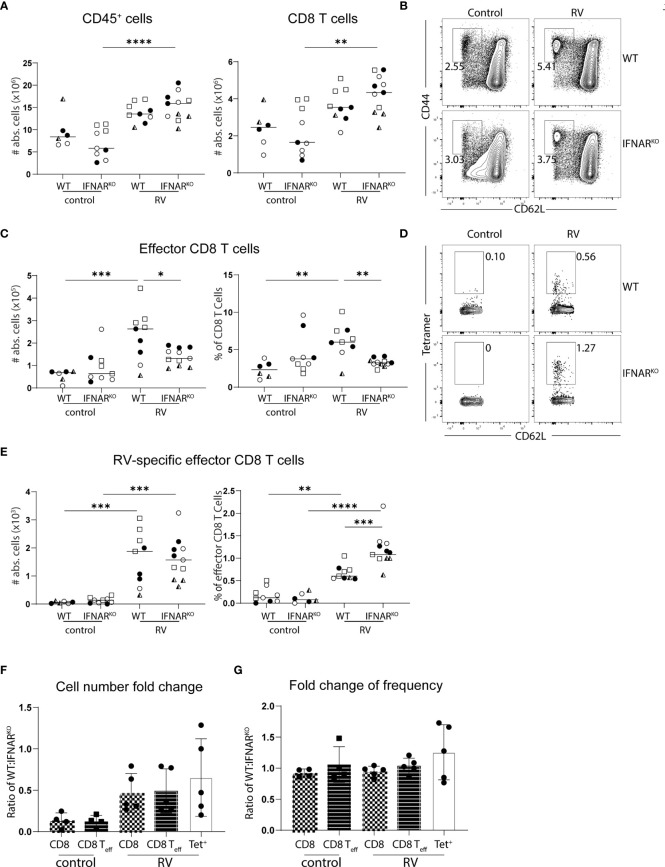
Global deficiency of IFNAR leads to a loss of effector CD8 T cells without altering the RV-specific CD8 T response. WT and IFNAR^KO^ mice were infected with RV. **(A–G)** MLN cells isolated from uninfected control and RV infected WT and IFNAR^KO^ mice seven days post oral infection. **(A)** Total number of CD45^+^ cells and CD8 T cells (CD45^+^CD3^+^CD4^-^CD8α^+^). **(B)** Representative FACS plot of effector CD8 T cells (CD62L^-^CD44^+^) pre-gated on CD45^+^CD3^+^CD4^-^CD8α^+^ cells. **(C)** Absolute cell numbers and frequency of effector CD8 T cells (CD62L^-^CD44^+^). **(D)** Representative FACS plot of RV-specific effector CD8 T cells (CD44^+^CD62L^-^Tet^+^). **(E)** Absolute cell numbers and frequency of RV-specific effector CD8 T cells. Each data point represents one mouse from a total of four different experiments, indicated by the different symbols. Ordinary one-way ANOVA with Tukey´s multiple comparison test was performed for statistical analysis. *p < 0.05, **p < 0.01, ***p < 0.001, ****p < 0.0001. Fold change of **(F)** absolute cell number and **(G)** frequency of indicated cells in mixed bone marrow chimeras of 50:50 WT : IFNAR^KO^ into WT recipients. Each data point represents one mouse from 2 independent experiments with 2-3 mice per group. Ordinary one-way ANOVA with Tukey´s multiple comparison test was performed for statistical analysis. Data not significant.

Type I IFNs are the major coordinators of anti-viral responses, so we next assessed what role global type I IFN signaling might play in inducing RV-specific CD8 T cells. To this end, we analyzed the immune response of complete type I IFN receptor (IFNAR)-deficient mice to RV infection. As we reported previously (21), MLN hypertrophy was not impacted by the absence of type I IFN signaling, resulting in a comparable total CD45^+^ and CD8 T cell number increase between IFNAR^KO^ and WT control mice at seven days post-infection (**Figure 4A**). In contrast, IFNAR^KO^ mice showed a selective decrease in the frequency and numbers of CD62L^-^CD44^+^ effector CD8 T cells (**Figures 4B, C**). Surprisingly, this did not translate to a deficiency in RV-specific CD8 T cell immunity, as the frequency of RV-specific cells among effector CD8 T cells was significantly enhanced in IFNAR^KO^ mice, leading to overall comparable RV-specific CD8 T cell numbers between IFNAR^KO^ and WT control mice (**Figures 4D, E**).

To test whether type I IFN supports RV-specific CD8 T cells in a competitive setting, we generated mixed bone-marrow (BM) chimeras by reconstituting irradiated WT recipients with a 50:50 mix of WT- and IFNAR^KO^-derived BM. To our surprise, we consistently found that IFNAR^KO^-derived BM had a striking reconstitution advantage over WT BM across all populations quantified (CD45^+^, CD8^+^, CD62L^-^CD44^+^ effector CD8), a currently inexplicable finding inconsistent with the literature. Interestingly, this effect somewhat levelled out following RV infection, suggesting that type I IFN contributes to RV infection-induced MLN hypertrophy in a cell intrinsic manner (**Figure 4F**). This differs from the non-competitive full IFNAR^KO^ scenario, in which MLN hypertrophy in response to RV infection is not impacted (21). Nevertheless, the frequency of IFNAR^KO^ and WT BM-derived RV-specific CD8^+^ effector T cells was comparable, suggesting that there is no intrinsic requirement for type I IFN for the accumulation of RV-specific CD8 T cells (**Figure 4G**).

**Figure 5:**
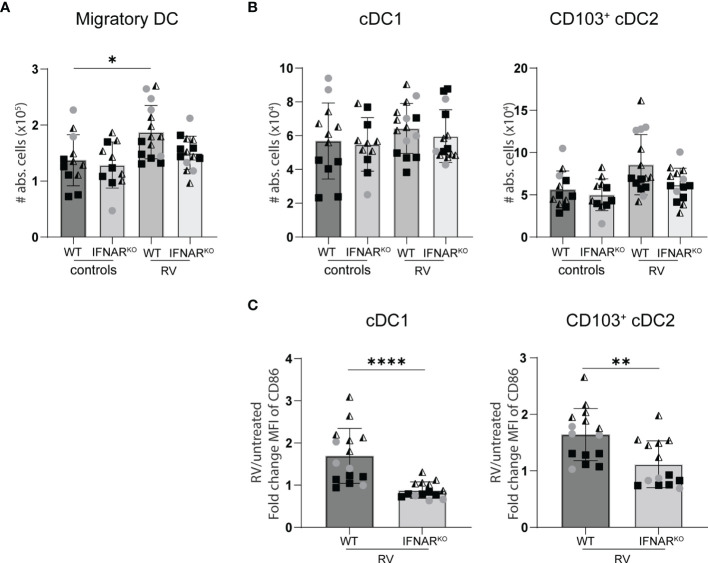
Lack of IFNAR signaling did not alter the migration of dendritic cell to the small intestine draining MLN. WT and IFNAR^KO^ mice were infected with RV and four days post oral infection the small intestine draining MLN were collected and analysed by flow cytometry. **(A)** Absolute cell number of migratory DCs (Lineage^-^CD64^-^CD11c^+^MHCII^high^). **(B)** Absolute number of cDC1 (CD103^+^CD11b^-^) and cDC2 (CD103^+^CD11b^+^). **(C)** Fold change of MFI of CD86 from cDC1 and cDC2 from RV-infected mice versus WT control mice. Ordinary one-way ANOVA with Tukey´s multiple comparison test was performed for statistical analysis of **(A**, **B)**. Mann Whitney U test was used for panel **(C)**. *p < 0.05, **p < 0.01, ***p < 0.001, ****p < 0.0001.

We next investigated whether effector functions of CD8 T cells were affected by the absence of global type I IFN signaling. In line with what we detected in MLN (**Figure 4E**), IFNAR^KO^ mice harboured equal numbers of RV-specific CD8 T cells in the SILP seven days post infection (**Figure 5A**) and 14 days post infection (**Figure 5B**), suggesting that type I IFN signaling was dispensable for CD8 effector T cell localization to, and maintenance at, the effector site. Absence of type I IFN signaling however caused a deficiency in the ability of MLN effector CD8 T cells to produce IFNγ, an effect already apparent at steady state and further pronounced in the context of RV infection (**Figure 5C**). PMA/Ionomycin and RV-peptide specific restimulation revealed similar dependencies of peptide-specific and other effector CD8 T cells on type I IFN-signaling (**Figure 5C**). To further assess functional consequences of IFNAR deficiency, we assessed surface expression of CD107a/b on RV-peptide restimulated CD8 T cells as a marker for degranulation capacity (26) and expression of granzyme A in RV-specific and bystander CD8 T cells. Indeed, significantly fewer RV-specific effector CD8 T cells from IFNAR-deficient mice expressed CD107a/b (**Figure 5D**). RV infection significantly increased the fraction of CD8 effector T cells expressing granzyme A in a type I IFN dependent manner, regardless of whether they stained positive for RV-tetramer or not (**Figure 5E**). Nevertheless, and in accordance with similar RV-specific CD8 T cell numbers in MLN and SILP, clearance of RV infection was equally effective in IFNAR^KO^ and WT control mice despite a higher viral load in IFNAR^KO^ mice early upon infection (**Figure 5F**).

**Figure 6:**
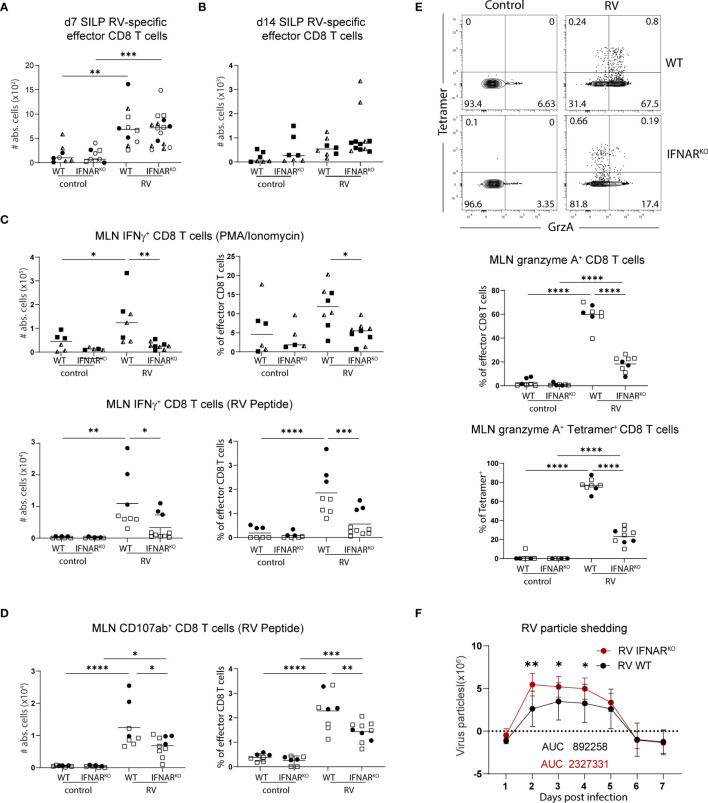
Global deficiency in type I IFN signaling does not affect localisation of RV-specific effector CD8 T cells but impacts IFNγ expression of bulk CD8 T cells. WT and IFNAR^KO^ mice were orally infected with RV. **(A)** Absolute numbers of RV-specific effector CD8 T cells (CD45^+^CD3^+^CD4^-^CD8α^+^CD44^+^Tet^+^) from SILP seven days post infection and **(B)** two weeks post infection. **(C)** MLN cells isolated seven days post infection and restimulated with PMA/Ionomycin (top panels) and MLN cells isolated five days post infection and restimulated with RV peptide (bottom panels). Absolute cell number and frequency of IFNγ positive CD8 T cells (CD45^+^CD3^+^CD4^-^CD8α^+^CD44^+^IFNγ^+^) analysed by flow cytometry. **(D)** MLN cell isolated five days post infection and absolute cell number and frequency of CD107αβ positive cells (CD45^+^CD3^+^CD4^-^CD8α^+^CD44^+^CD107ab^+^) analysed by flow cytometry after RV peptide restimulation. **(E)** FACS plots are pre-gated on effector CD8 T cells from MLN cells isolated five days post infection. Frequency of total granzyme A^+^ (CD45^+^CD3^+^CD4^-^CD8α^+^CD44^+^GranzymeA^+^) amongst effector CD8 T cells and tetramer^+^ granzyme A^+^ cells of total tetramer^+^ effector CD8 T cells are shown. **(F)** Level of RV shedding measured by ELISA on fecal samples collected at indicated time points. **(A–F)** Each data point represents one mouse from a total of two **(B–E)** or four **(A, F)** different experiments, indicated by the different symbols. Ordinary one-way ANOVA with Tukey´s multiple comparison test was performed for statistical analysis. *p < 0.05, **p < 0.01, ***p < 0.001, ****p < 0.0001.

Similar RV-specific CD8 T cell priming in IFNAR^KO^ and WT control mice suggests equal antigen presentation. We therefore quantified the RV-induced migration of intestinal cDC1 (CD103^+^CD11b^-^) and cDC2 (CD103^+^CD11b^+^) to the small intestine-draining MLN (sMLN) four days post infection, the only time point we found to consistently show an increase of cDC numbers in the sMLN in response to RV infection (**Figure 6A**). IFNAR-deficiency did not significantly impact on the migration of either cDC1 or cDC2 to the sMLN (**Figure 6B**). However, both subsets showed significant defects in their ability to upregulate the co-stimulatory molecule CD86 in IFNAR^KO^ mice (**Figure 6C**), explaining the diminished overall effector CD8 T cell induction in response to RV infection in IFNAR^KO^ mice and possibly contributing to qualitative defects as assessed by IFNγ and granzyme production and their ability to display CD107a/b on their surface upon antigen-specific restimulation.

## Discussion

We here describe that the individual absence of the innate immune signaling pathways governed by TLR3/TRIF, MyD88 and the type I IFN receptor does not affect CD8 T cell induction in response to asymptomatic RV infection in adult mice. This is surprising since TLR3, MyD88 and type I IFN were all implied in several aspects of immune protection from RV infection before.

TLR3 is highly conserved across species and exerts non-redundant functions in the context of several viral infections across mice and humans (reviewed in (27)). Adult mice deficient in TLR3 were previously shown to be more susceptible to RV with a higher RV burden at the peak of infection (19). In this study, TLR3 expression on both hematopoietic and non-hematopoietic cells contributed to viral defense. A previous study using a heterologous RV strain reported that TLR3/TRIF sensing by the epithelium was redundant, and that innate epithelial immunity instead depended on RIG-I/MAVS signaling (28). Using the highly infectious homologous RV strain EC_W_, we found that TLR3-deficiency did not affect the viral load upon RV infection in our colony. Human studies revealed that TLR3 drives basal type I IFN production by fibroblasts, while RIG-I/MDA5-mediated dsRNA recognition induces acute virus-induced type I IFN production (29, 30). Steady state type I IFN signaling in the intestines is required for a “basal poised state” in intestinal DCs, endowing them with the ability to induce adaptive immunity (25). However, steady state type I IFN induction by bacteria may differ depending on the microbial context. This could explain why, using specific mouse strains in a specific microbial housing context, TLR3 may contribute to early viral containment in some cases, but not in others, matching the low clinical penetrance observed in humans carrying mutations within TLR3 (30).

We also found unaltered RV-specific CD8 T cell responses in TLR3-deficient mice, matching the similar shedding kinetics. TLR3 was previously shown to promote cross-priming of CD8 T cells to virus-infected cells (31) and is highly expressed on cDC1 and required for their migration to the MLN in the context of poly(I:C) stimulation (20). Nevertheless, we here report that TLR3 is redundant for the induction of cytotoxic immunity to RV, suggesting that other sensing pathways can compensate for the lack of TLR3 in this context.

All TLRs except TLR3 signal through MyD88, and TLR7 has previously been implicated in the production of type I IFN and TNFα in plasmacytoid DCs (pDCs) in response to RV infection (32). Depletion of pDCs leads to enhanced shedding of RV (33). In addition, MyD88 mediates IL-1 and IL-18 signaling downstream of the IL-1 receptor and inflammasome signaling plays a role in early containment of the viral infection (34, 35). In contrast to a study conducted by Uchiyama et al. (23), who showed that early control of RV loads depended on the role of MyD88, we found that mice deficient in MyD88 and WT controls shed comparable amounts of RV in their feces at early time points after infection. These discrepancies are possibly due to the different microbial status between animal facilities, a variable previously shown to affect RV infectivity, which was significantly dampened in segmented filamentous bacteria (SFB)-harboring RAG-deficient mice due to blooming of the strain in absence of adaptive immune control (36). Epithelial cell-specific MyD88 deficiency was reported to not alter SFB loads (37) and we confirmed this finding in complete MyD88^KO^ mice in our colony (**Supplementary Figure 1**). Nevertheless, the microbiome constitution is known to regulate RV infectivity [reviewed in (38)], suggesting that selected microbes overrepresented in MyD88-deficient mice in our facility may account for the absence of an overall higher RV burden in our mice.

In addition to its role in epithelial restriction of viral replication, MyD88-deficiency led to a significantly higher RV-specific IgG1 over IgG2c ratio due to abrogated IL-1/IL-18 signaling, effector molecules of inflammasome pathways that signal through MyD88 (23). In contrast, we found that MyD88 was dispensable for RV-specific CD8 T cell induction and accumulation. In fact, those cells were slightly elevated in MyD88-deficient mice, possibly caused by the general decreased barrier integrity in the absence of MyD88 (39). These findings are surprising, as CD8 T cell-intrinsic MyD88 signaling was shown to be required for their maintenance upon LCMV infection (40). LCMV is a systemic and much stronger inducer of T cell immunity than RV, possibly accounting for the differences in T cell-intrinsic requirements for survival upon infection.

Using intraperitoneal injection of the dsRNA mimic poly(I:C), it was previously shown that almost all cellular changes imposed by TRIF/MyD88/MAVS-mediated signaling in DCs are secondary through autocrine and paracrine type I IFN signaling (24). Accordingly, IFNAR-deficient DCs are unable to support TH1 cell differentiation upon vaccination with an HIV gag protein vaccine adjuvated with poly(I:C) (41). We have previously shown that IFNAR-deficient cDC1 are unable to migrate from the intestines to the MLN in response to poly(I:C) injection, a process that requires sensing by TLR3 (20). Conversely, we here report that antigen-specific CD8 T cell induction in response to RV infection, a process that at least partially depends on cDC1 (6, 7), occurs independently of type I IFN and TLR3 and that neither pathway is required for clonal expansion during the primary response in the MLN. The redundancy of IFNAR-signaling is particularly surprising given that many of the major virus-sensing pathways converge in this pathway, including RIG-I/MDA5 and TLR7 and that we have previously shown that plasmacytoid-derived type I interferon facilitates early B cell activation in response to RV (33). In addition, we found that upregulation of the costimulatory molecule CD86 in response to RV infection clearly depended on type I IFN signaling. In contrast to a similar expansion of RV-specific CD8 T cells in the absence of type I IFN signaling, we detected significant deficiencies in their ability to express granzyme, to produce IFNg and to display CD107a/b on their surface. upon antigen-specific restimulation. Nevertheless, clearance of the virus, previously shown to depend on CD8 T cells (18), occurred normally in IFNAR-deficient mice. This suggests that the residual responses occurring in the absence of type I IFN signaling suffice for viral clearance, or that cytotoxic pathways not assessed in our study remain intact. It will therefore be of interest to investigate in the future whether the quality of CD8 T cell priming differs in presence or absence of type I IFN with regards to cytotoxicity or memory induction. Along those lines, a previous study using respiratory syncytial virus infection revealed similar kinetics of CD8 T cell induction in MAVS^KO^ mice, but a deficiency in CD8 memory recall upon reinfection (42, 43). The fact that primary RV infection completely protects from reinfection in mice by inducing a strong long-lasting RV-specific IgA response however prevents the assessment of CD8 T cell memory upon natural reinfection in this model.

Type I IFN signaling can also affect cytotoxic T cell induction, functionality, and survival in a direct manner, and this seems highly context dependent. Type I IFN signaling in human T cells was shown to enhance activation-induced cell death (AICD) through upregulation of FAS and FAS ligand expression, leading to increased cell death (44). Likewise, type I IFN early after infection was reported to cause a loss particularly of bystander CD44^hi^ memory T cells in T cell receptor (TCR) transgenic mice bearing an irrelevant TCR (45). In the context of Listeria infection, type I IFN was shown to sensitize lymphocytes to bacteriolysin-mediated cell death early after infection independently of IL-1, IL-12, IFNγ, FAS, or reactive oxygen or nitrogen species, and IFNAR-deficient mice controlled the infection better than WT control mice (46). Conversely, type I IFN was previously shown to enhance CD8 cell proliferation and memory formation upon stimulation with strong inducers of type I IFN (47, 48). We here show that in the context of RV infection, total CD8 T cell numbers and RV-specific CD8 T effector cell accumulation in the MLN seven days post-infection were comparable in IFNAR^KO^ and WT control mice, while total effector CD8 T cell numbers were significantly lower in the absence of IFNAR signaling. As naïve CD8 T cells and effector CD8 T cells from IFNAR^KO^ and WT control donors contributed with equal ratios to the response in mixed BM chimeras, the deficiency in non-RV specific CD8 effector T cell priming is a cell-extrinsic event, possibly caused by blunted DC activation in the absence of IFNAR-signaling. Our findings suggest the existence of an IFNAR-independent backup system of presumably other cytokines to assist virus-specific CD8 T cell responses against poor type IFN inducers such as homologous RV. Nevertheless, the expression of IFNγ and granzyme, classical effector CD8 T cell hallmarks, are blunted in the absence of type I IFN signaling and this affects RV-specific and bystander responses alike.

Together, we here describe a remarkable lack of consequence on the induction of RV-specific CD8 T cell responses caused by the individual absence of innate signaling pathways, which differs from previous findings in studies using model adjuvants. Homologous RV infection in adult mice triggers measurable CD8 T cell immunity and serves as a useful model to study the connection of innate immune sensing pathways in inducing cellular immunity to an asymptomatic enteric viral infection. This has implications for both vaccination strategies as well as for the better understanding of immune homeostasis at the intestinal barrier, which is constantly exposed to eukaryotic viruses even at steady state.

## Material and Methods

### Mice

All animals were housed under specific pathogen-free conditions at Lund/Malmö, Sweden or at Kongens Lyngby, Denmark. All mice were on the C57BL/6 background (B6.SJL-PtprcaPepcb/BoyJ for CD45.1 BM donors). Age-matched male and female mice between 8 and 16 weeks were used. IFNAR^KO^ mice (B6(Cg)-ifnar^tm1.2Ees^/J) were purchased from the Jackson Laboratory and bred and maintained in the Clinical Research Center at Lund University or the BioX facility at the Danish Technical University. XCR1.IFNAR^KO^ were generated by crossing B6-Xcr1tm2Ciphe mice (49) to IFNAR floxed mice [obtained from U. Kalinke (50)]. We used TLR3^LSL/LSL^ (51) and MyD88^LSL/LSL^ (52) mice, which are deficient for TLR3 and MyD88, respectively, in the absence of cre recombinase. Animal experiments were performed under appropriate licenses within local and national guidelines for animal care.

### Rotavirus Infection

The virulent WT EC_w_ strain of RV was obtained from RV infected 5 days old suckling mice. Two-day post infection the intestines were collected, and intestinal homogenates were prepared and used for infection. Adult mice were inoculated orally at a dose of 3 × 10^3^ diarrhea-inducing dose 50 (DD_50_) and the mice were sacrificed five, seven or 14 days post infection for analysis.

### Cell Isolation

MLN lymphocytes were obtained by mechanical disruption and filtered through a 70μm cell strainer in staining buffer [2% FBS (Sigma), 1mM EDTA (Invitrogen), 500ml PBS (GIBCO)]. DC isolation from the small intestinal draining MLN was performed by digestion with collagenase IV (0.5 mg/mL, Sigma–Aldrich) and DNase I (12.5 μg/mL, Roche) diluted in R10 media (RPMI 1640 + 10% FCS) for 40 min at room temperature, followed by mechanical disruption and filtering through a 70μm cell strainer. For the isolation of small intestinal lamina propria (SILP) lymphocytes, the intestine was opened longitudinally after removal of Peyer’s Patches and fat and cut into small pieces. Tissue pieces were incubated on a shaker in HBSS containing 2% FBS and 2mM EDTA at 37°C for three rounds (first round 10 mins, followed by two rounds of 15 mins each). Buffer exchange was performed by discarding the supernatant through a nylon mesh, retaining the tissue pieces. Subsequently, the remaining tissue was digested in RPMI medium (Gibco) containing 10% FBS, 58μg/ml Liberase TM (Roche) (amounting to 0.3 WünschU/ml) and 30μg/ml DNase I (Roche) on a magnetic stirrer for 20 mins at 37°C. Upon digestion, the cell suspension was filtered through a 100μm strainer (Fisher Scientific) and lymphocytes were enriched by density gradient centrifugation with 40%/70% Percoll (GE Healthcare).

### Flow Cytometry

For surface T cell staining, nonspecific binding was blocked with 10% rat serum (Sigma) and rat α-mouse CD16/CD32 Fc block (2.4G2) for 20 min at 4°C. Dead cells were excluded using propidium iodide (Sigma–Aldrich) and cell aggregates were excluded from the analysis by identification on FSC-A versus FSC-H scatterplots. The following antibodies were used for the identification of RV-specific CD8 T cells by flow cytometry: BV510 α-CD45 (30-F11), FITC α-CD3 (17A2), APC α-CD62L (MEL-14), PE-Cy7 α-CD44 (IM7), PE α-CD8α (53-6.7), and APC-CY7 α-CD4 (RM4-5). RV-specific CD8 T cells were identified using BV421-labeled tetramer containing the RV VP6_VGPVFPPGM_ immunodominant peptide.

For DC staining, dead cells were stained using Fixable Viability Dye eFlour™-780 (ThermoFisher Scientific) according to the manufacturer’s instructions. For subsequent staining procedures, Ca/Mg-containing PBS supplemented with 2% FCS was used. Non-specific binding was blocked with rat α-mouse CD16/CD32 Fc-block (93) for 20 min at 4°C. DCs were identified by using the following antibodies: PE-Cy7 α-CD3 (145-2C11), PE-Cy7 α-CD19 (eBio1D3), PE-Cy7 α-NK.1.1 (PK136) PE-Cy7 α-B220 (RA3-6B2), PE-Cy7 α-CD64 (X54-5/7.1), BUV395 α-CD45 (30-F11), BV421 α-CD11c (N418), BV510 α-MHC-II (M5/114), PE α-XCR1 (LET), BV605 α-CD11b (M1/70) and APC α-CD86 (GL1).

Experiments were designed in line with the published guidelines for flow cytometry (53). T cell data were acquired on a LSRII and DC data were acquired on a Fortessa (both BD Biosciences) and analyzed using FlowJo software 10. For CD86 MFI fold change calculation, we first subtracted the FMO MFI value from all values, followed by averaging all PBS control values within the same experiment. RV-treated values are depicted as fold change over the resulting average PBS-control values.

### T Cell Stimulation

Control and RV infected mice were injected with 20 µg of FTY720 (Sigma Aldrich) every other day starting from day 1 after RV infection. Seven days post infection for PMA/Ionomycin or 5 days post infection for RV peptide restimulation, MLN lymphocytes were isolated as described above and subjected to *in vitro* stimulation: 10-12*10^6^ cells were stimulated with either 250 µg/ml phorbol 12-myristate 13-acetate (PMA) (Sigma Aldrich) and 500 µg/ml ionomycin (Sigma Aldrich) or 2µg/ml of RV Peptide (VGPVFPPGM) (JPT peptide Technologies) in a total volume of 1ml complete medium (RPMI (Gibco) with 10% FBS) at 37°C for a total of 4 and 6 hours, respectively. 1µl of Golgi stop (BD Bioscience) was added after one hour of stimulation with PMA/Ionomycin, or at the beginning of the restimulation with RV peptide. Cells were stained for surface markers using PE CD8α (53-6.7), BV510 CD45 (30-F11) or A700 CD45 (104), A700 CD3 (17-A2) or PE-CF594 TCRβ (H57-597), FITC CD4 (RM4-5), PE-CY7 CD44 (IM7), APC-CY7 CD62L (MEL-14), A647 CD107a (1D4B), and A647 CD107b (M3/84). The cells were then fixed and permeabilized using the Foxp3/Transcription Factor Staining Buffer Set (eBioscience) according to the manufacturer’s instructions, followed by intracellular staining using BV605 IFNγ (XMG1.2) and PerCP-eFluor 710 Granzyme A (GzA-3G8.5).

### RV Shedding ELISA

For measurement of RV antigen, fecal samples collected daily from RV infected and uninfected controls over 7 days were weighed and soaked in PBS containing 1% BSA, 1mM EDTA, soybean trypsin inhibitor (0.05 mg/ml), 2mM PMSF (phenylmethylsulfonyl fluoride) (Sigma) and 0.025% sodium azide for 2 hours at a concentration of 100 mg/ml, followed by mechanical homogenization and centrifugation at 13,000rpm for 10 minutes. The supernatants were collected and stored for RV particle shedding ELISA. Soft vinyl round bottom ELISA plates (Fisher scientific, Ref# 10324165) were coated with guinea pig anti-RV hyperimmune serum (gift from Prof. Harry Greenberg) at 1:5000 in PBS and incubated at 37°C for 4hrs followed by blocking with 2% BSA in PBS for 2hrs at 37°C. Subsequently, fecal samples were diluted 1:20 in 0.5% BSA and added to the ELISA plate followed by incubation at 4°C overnight. Rabbit anti-RV hyperimmune serum (gift from Prof. Harry Greenberg) was added (1:5000 in PBS) and incubated for 2hrs at 37°C. Detection was performed using α-rabbit HRP antibody (BD pharmingen) for 1hr at 37°C at 1:12000 followed by TMB substrate (BD OptEIA) addition to visualize the reaction. A known concentration of resus rotavirus was used as a standard for quantification.

### Bone Marrow Chimera Setup

Mixed BM chimeras were generated by sublethal irradiation (9 Gy) of CD45.1/2 mice followed by reconstitution with 6 X 10^6^ BM cells at the ratio of 50:50 WT: IFNAR^-/-^ intravenously. Analysis of BM chimeras was performed 7-8 weeks after BM transfer. WT-derived cells were identified by staining for the congenic marker CD45.1 and IFNAR^-/–^derived cells by staining for CD45.2 during analysis.

### RT-PCR for SFB

Bacterial genomic DNA isolation from fecal samples was performed using the QIAamp PowerFecal DNA Kit (QIAGEN) following the manufacturer’s instructions. Real-time PCR was performed using a MyiQ BioRad Real Time System and SYBR Green detection (BioRad) with SFB primers: 5’-AGGAGGAGTCTGCGGCACATTAGC (forward) and 5’-TCCCCACTGCTGCCTCCCGTAG (reverse) (54). For the SYBR green-based PCR, 8 ng of gDNA was used as template and primer concentrations were 300 nM. Reactions were run for 10 min at 95°C followed by 35 cycles of 20 sec at 65°C, 20 sec at 72°C, and 15 sec at 95°C. Samples were assayed in triplicates and gene expression levels for each sample were normalized relative to internal 16S rRNA gene detection with ΔCt calculation.

### Statistical Analysis

Graph pad prism 9 software was used for statistical analysis. Statistics were performed using one-way ANOVA with Tukey´s multiple comparison test.

## Data Availability Statement

The original contributions presented in the study are included in the article/**Supplementary Material**. Further inquiries can be directed to the corresponding author.

## Ethics Statement

The animal study was reviewed and approved by Malmö - Lunds djurförsöksetiska nämnd #04525/2017.

## Author Contributions

KGM, IU, KH, SD, and JN performed and analyzed experiments. KGM and KL conceived the study and wrote the manuscript. All authors read the manuscript and contributed with final edits. All authors contributed to the article and approved the submitted version.

## Funding

KL was supported by the Lundbeck Foundation (Fellowship R215-2015-4100), the Ragnar Söderberg Foundation (Fellowship in Medicine), Vetenskapsrådet (2020-01977), the Crafoord Foundation, Gyllenstiernska Krapperupsstiftelsen, the Julin Foundation, the Apotekare Hedberg Foundation, and the Birgitta och Göran Karlsson Foundation. KL, KGM, and JN received project funding from the Kungliga Fysiografiska Sällskapet Lund.

## Conflict of Interest

The authors declare that the research was conducted in the absence of any commercial or financial relationships that could be construed as a potential conflict of interest.

## Publisher’s Note

All claims expressed in this article are solely those of the authors and do not necessarily represent those of their affiliated organizations, or those of the publisher, the editors and the reviewers. Any product that may be evaluated in this article, or claim that may be made by its manufacturer, is not guaranteed or endorsed by the publisher.

## Supplementary Material

Supplementary Figure 1 WT control and MyD88^KO^ mice are colonized equally with SFB. Fecal pellets from 3 WT control and 5 MyD88^KO^ mice were sampled and tested for ribosomal SFB RNA, normalized to rRNA detected using universal 16S primers, by RT-PCR.
