# Supplementary figures and images for: Rotavirus-Induced Expansion of Antigen-Specific CD8 T Cells Does Not Require Signaling via TLR3, MyD88 or the Type I Interferon Receptor
Source: Front Immunol. 2022 Apr 7;13:814491. doi: 10.3389/fimmu.2022.814491 (PMC9022177; doi:10.3389/fimmu.2022.814491)

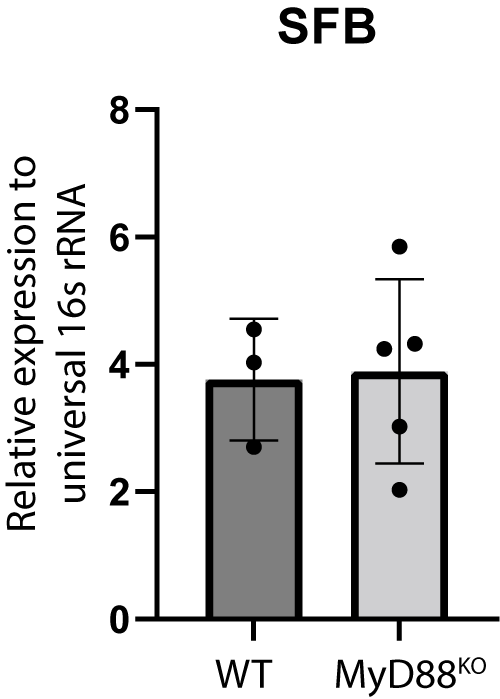

Supplement: Supplementary Figure 1 — WT control and MyD88KO mice are colonized equally with SFB. Fecal pellets from 3 WT control and 5 MyD88KO mice were sampled and tested for ribosomal SFB RNA, normalized to rRNA detected using universal 16S primers, by RT-PCR. [file Image_1.tif]
